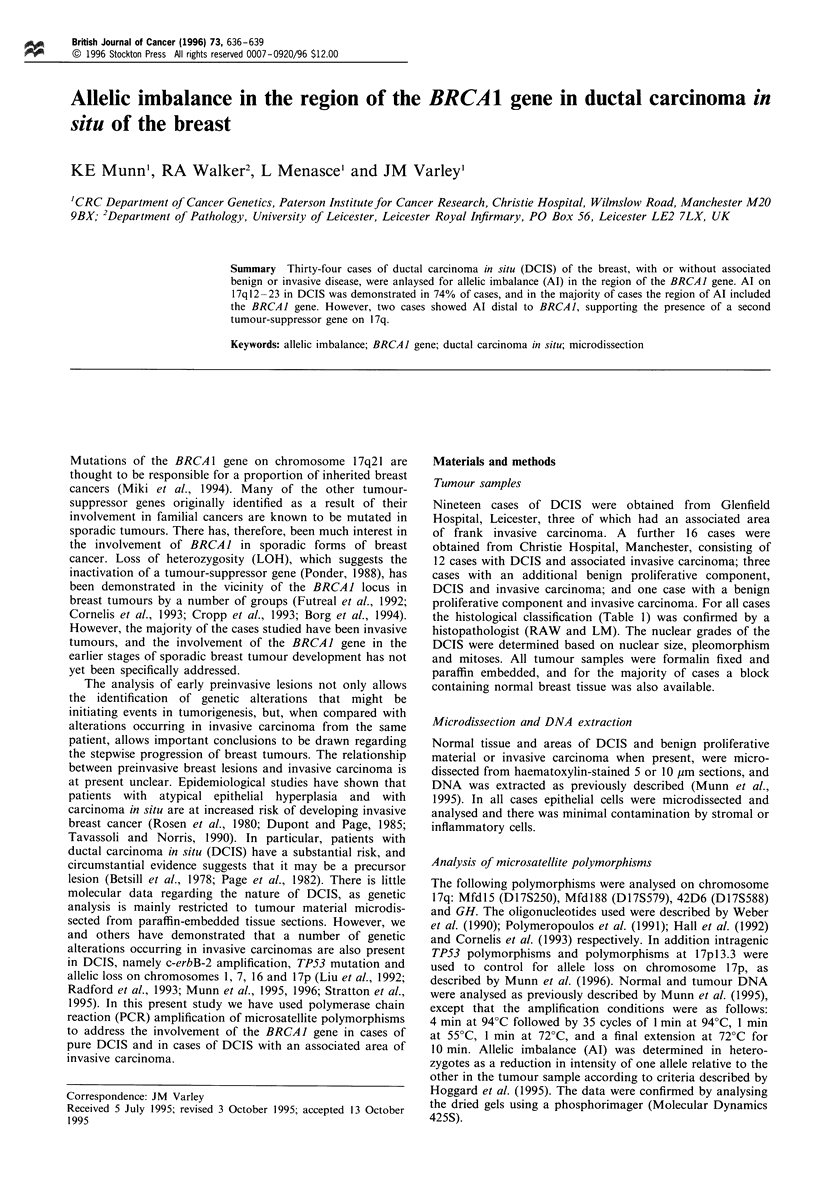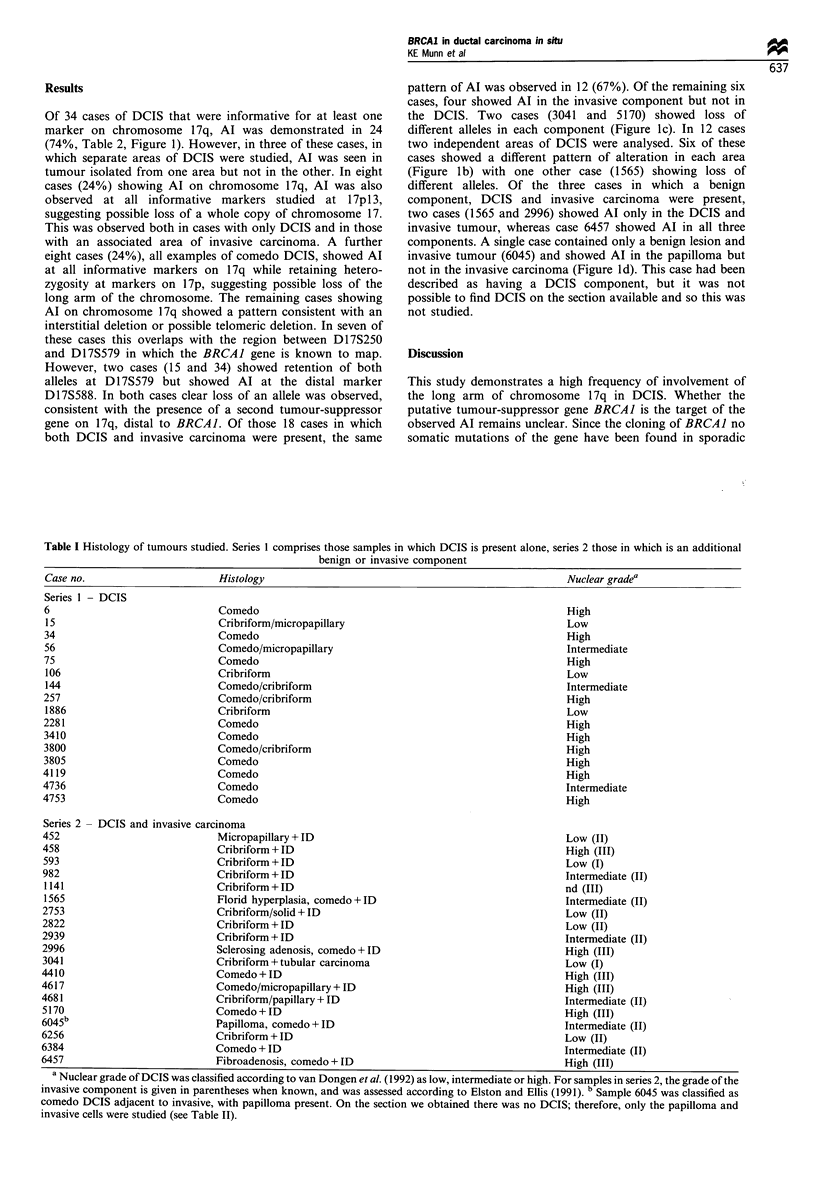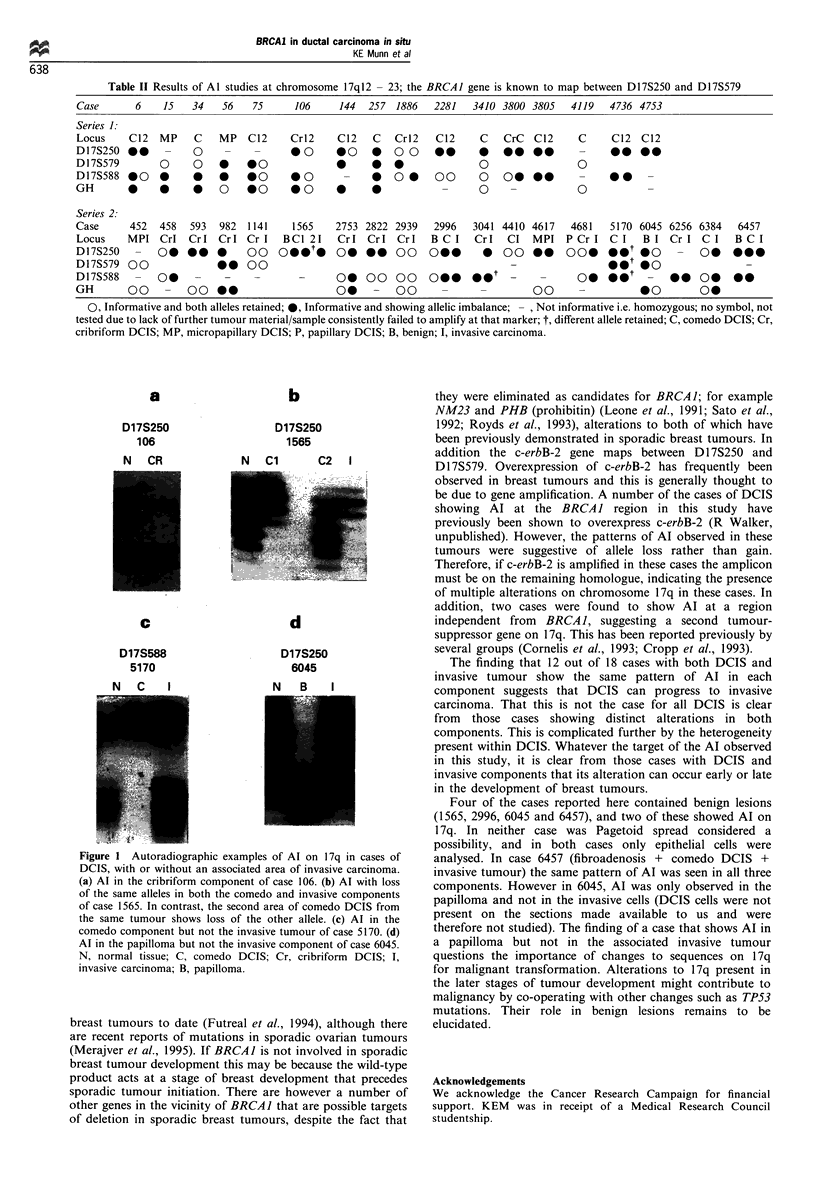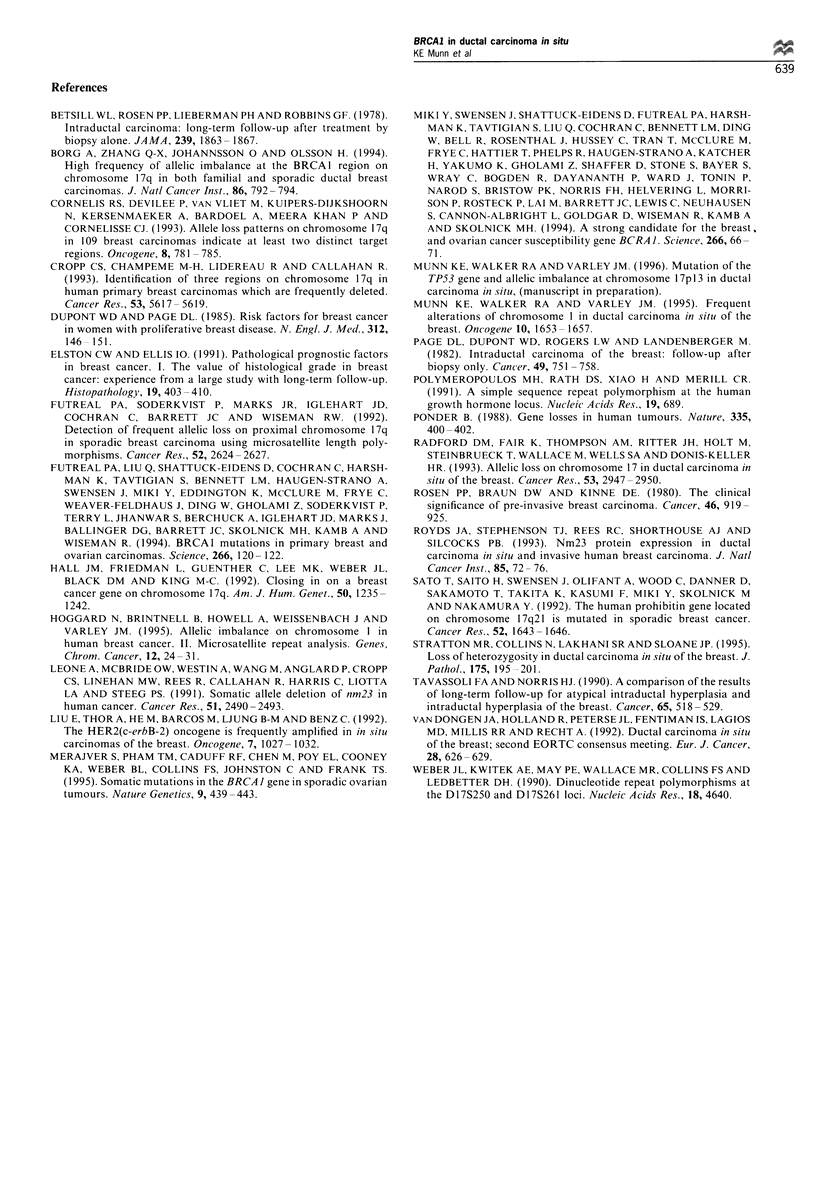# Allelic imbalance in the region of the BRCA1 gene in ductal carcinoma in situ of the breast.

**DOI:** 10.1038/bjc.1996.110

**Published:** 1996-03

**Authors:** K. E. Munn, R. A. Walker, L. Menasce, J. M. Varley

**Affiliations:** CRC Department of Cancer Genetics, Paterson Institute for Cancer Research, Christie Hospital, Manchester, UK.

## Abstract

**Images:**


British Journal of Cancer (1996) 73, 636-639
© 1996 Stockton Press All rights reserved 0007-0920/96 $12.00

### Allelic imbalance in the region of the *BRCA*1 gene in ductal carcinoma *in situ* of the breast

KE Munn[1], RA Walker[2], L Menasce[1] and JM Varley[1]

[1]*CRC Department of Cancer Genetics, Paterson Institute for Cancer Research, Christie Hospital, Wilmslow Road, Manchester M20 9BX;* [2]*Department of Pathology, University of Leicester, Leicester Royal Infirmary, PO Box 56, Leicester LE2 7LX, UK*

**Summary** Thirty-four cases of ductal carcinoma *in situ* (DCIS) of the breast, with or without associated benign or invasive disease, were anlaysed for allelic imbalance (AI) in the region of the *BRCA1* gene. AI on 17q12-23 in DCIS was demonstrated in 74% of cases, and in the majority of cases the region of AI included the *BRCA1* gene. However, two cases showed AI distal to *BRCA1*, supporting the presence of a second tumour-suppressor gene on 17q.

**Keywords:** allelic imbalance; *BRCA1* gene; ductal carcinoma *in situ*; microdissection

Mutations of the *BRCA*1 gene on chromosome 17q21 are thought to be responsible for a proportion of inherited breast cancers (Miki *et al.*, 1994). Many of the other tumour-suppressor genes originally identified as a result of their involvement in familial cancers are known to be mutated in sporadic tumours. There has, therefore, been much interest in the involvement of *BRCA1* in sporadic forms of breast cancer. Loss of heterozygosity (LOH), which suggests the inactivation of a tumour-suppressor gene (Ponder, 1988), has been demonstrated in the vicinity of the *BRCA1* locus in breast tumours by a number of groups (Futreal *et al.*, 1992; Cornelis *et al.*, 1993; Cropp *et al.*, 1993; Borg *et al.*, 1994). However, the majority of the cases studied have been invasive tumours, and the involvement of the *BRCA1* gene in the earlier stages of sporadic breast tumour development has not yet been specifically addressed.

The analysis of early preinvasive lesions not only allows the identification of genetic alterations that might be initiating events in tumorigenesis, but, when compared with alterations occurring in invasive carcinoma from the same patient, allows important conclusions to be drawn regarding the stepwise progression of breast tumours. The relationship between preinvasive breast lesions and invasive carcinoma is at present unclear. Epidemiological studies have shown that patients with atypical epithelial hyperplasia and with carcinoma *in situ* are at increased risk of developing invasive breast cancer (Rosen *et al.*, 1980; Dupont and Page, 1985; Tavassoli and Norris, 1990). In particular, patients with ductal carcinoma *in situ* (DCIS) have a substantial risk, and circumstantial evidence suggests that it may be a precursor lesion (Betsill *et al.*, 1978; Page *et al.*, 1982). There is little molecular data regarding the nature of DCIS, as genetic analysis is mainly restricted to tumour material microdissected from paraffin-embedded tissue sections. However, we and others have demonstrated that a number of genetic alterations occurring in invasive carcinomas are also present in DCIS, namely c-*erb*B-2 amplification, *TP53* mutation and allelic loss on chromosomes 1, 7, 16 and 17p (Liu *et al.*, 1992; Radford *et al.*, 1993; Munn *et al.*, 1995, 1996; Stratton *et al.*, 1995). In this present study we have used polymerase chain reaction (PCR) amplification of microsatellite polymorphisms to address the involvement of the *BRCA1* gene in cases of pure DCIS and in cases of DCIS with an associated area of invasive carcinoma.

Correspondence: JM Varley
Received 5 July 1995; revised 3 October 1995; accepted 13 October 1995

### Materials and methods

### *Tumour samples*

Nineteen cases of DCIS were obtained from Glenfield Hospital, Leicester, three of which had an associated area of frank invasive carcinoma. A further 16 cases were obtained from Christie Hospital, Manchester, consisting of 12 cases with DCIS and associated invasive carcinoma; three cases with an additional benign proliferative component, DCIS and invasive carcinoma; and one case with a benign proliferative component and invasive carcinoma. For all cases the histological classification (Table 1) was confirmed by a histopathologist (RAW and LM). The nuclear grades of the DCIS were determined based on nuclear size, pleomorphism and mitoses. All tumour samples were formalin fixed and paraffin embedded, and for the majority of cases a block containing normal breast tissue was also available.

### *Microdissection and DNA extraction*

Normal tissue and areas of DCIS and benign proliferative material or invasive carcinoma when present, were microdissected from haematoxylin-stained 5 or 10 $\mu$m sections, and DNA was extracted as previously described (Munn *et al.*, 1995). In all cases epithelial cells were microdissected and analysed and there was minimal contamination by stromal or inflammatory cells.

### *Analysis of microsatellite polymorphisms*

The following polymorphisms were analysed on chromosome 17q: Mfd15 (D17S250), Mfd188 (D17S579), 42D6 (D17S588) and *GH*. The oligonucleotides used were described by Weber *et al.* (1990); Polymeropoulos *et al.* (1991); Hall *et al.* (1992) and Cornelis *et al.* (1993) respectively. In addition intragenic *TP53* polymorphisms and polymorphisms at 17p13.3 were used to control for allele loss on chromosome 17p, as described by Munn *et al.* (1996). Normal and tumour DNA were analysed as previously described by Munn *et al.* (1995), except that the amplification conditions were as follows: 4 min at 94°C followed by 35 cycles of 1 min at 94°C, 1 min at 55°C, 1 min at 72°C, and a final extension at 72°C for 10 min. Allelic imbalance (AI) was determined in heterozygotes as a reduction in intensity of one allele relative to the other in the tumour sample according to criteria described by Hoggard *et al.* (1995). The data were confirmed by analysing the dried gels using a phosphorimager (Molecular Dynamics 425S).

### Results

Of 34 cases of DCIS that were informative for at least one marker on chromosome 17q, AI was demonstrated in 24 (74%, Table 2, Figure 1). However, in three of these cases, in which separate areas of DCIS were studied, AI was seen in tumour isolated from one area but not in the other. In eight cases (24%) showing AI on chromosome 17q, AI was also observed at all informative markers studied at 17p13, suggesting possible loss of a whole copy of chromosome 17. This was observed both in cases with only DCIS and in those with an associated area of invasive carcinoma. A further eight cases (24%), all examples of comedo DCIS, showed AI at all informative markers on 17q while retaining heterozygosity at markers on 17p, suggesting possible loss of the long arm of the chromosome. The remaining cases showing AI on chromosome 17q showed a pattern consistent with an interstitial deletion or possible telomeric deletion. In seven of these cases this overlaps with the region between D17S250 and D17S579 in which the *BRCA1* gene is known to map. However, two cases (15 and 34) showed retention of both alleles at D17S579 but showed AI at the distal marker D17S588. In both cases clear loss of an allele was observed, consistent with the presence of a second tumour-suppressor gene on 17q, distal to *BRCA1*. Of those 18 cases in which both DCIS and invasive carcinoma were present, the same pattern of AI was observed in 12 (67%). Of the remaining six cases, four showed AI in the invasive component but not in the DCIS. Two cases (3041 and 5170) showed loss of different alleles in each component (Figure 1c). In 12 cases two independent areas of DCIS were analysed. Six of these cases showed a different pattern of alteration in each area (Figure 1b) with one other case (1565) showing loss of different alleles. Of the three cases in which a benign component, DCIS and invasive carcinoma were present, two cases (1565 and 2996) showed AI only in the DCIS and invasive tumour, whereas case 6457 showed AI in all three components. A single case contained only a benign lesion and invasive tumour (6045) and showed AI in the papilloma but not in the invasive carcinoma (Figure 1d). This case had been described as having a DCIS component, but it was not possible to find DCIS on the section available and so this was not studied.

### Discussion

This study demonstrates a high frequency of involvement of the long arm of chromosome 17q in DCIS. Whether the putative tumour-suppressor gene *BRCA1* is the target of the observed AI remains unclear. Since the cloning of *BRCA1* no somatic mutations of the gene have been found in sporadic

**Table I** Histology of tumours studied. Series 1 comprises those samples in which DCIS is present alone, series 2 those in which is an additional benign or invasive component

| *Case no.* | *Histology* | *Nuclear grade*[a] |
|---|---|---|
| Series 1 – DCIS | | |
| 6 | Comedo | High |
| 15 | Cribriform/micropapillary | Low |
| 34 | Comedo | High |
| 56 | Comedo/micropapillary | Intermediate |
| 75 | Comedo | High |
| 106 | Cribriform | Low |
| 144 | Comedo/cribriform | Intermediate |
| 257 | Comedo/cribriform | High |
| 1886 | Cribriform | Low |
| 2281 | Comedo | High |
| 3410 | Comedo | High |
| 3800 | Comedo/cribriform | High |
| 3805 | Comedo | High |
| 4119 | Comedo | High |
| 4736 | Comedo | Intermediate |
| 4753 | Comedo | High |
| Series 2 – DCIS and invasive carcinoma | | |
| 452 | Micropapillary + ID | Low (II) |
| 458 | Cribriform + ID | High (III) |
| 593 | Cribriform + ID | Low (I) |
| 982 | Cribriform + ID | Intermediate (II) |
| 1141 | Cribriform + ID | nd (III) |
| 1565 | Florid hyperplasia, comedo + ID | Intermediate (II) |
| 2753 | Cribriform/solid + ID | Low (II) |
| 2822 | Cribriform + ID | Low (II) |
| 2939 | Cribriform + ID | Intermediate (II) |
| 2996 | Sclerosing adenosis, comedo + ID | High (III) |
| 3041 | Cribriform + tubular carcinoma | Low (I) |
| 4410 | Comedo + ID | High (III) |
| 4617 | Comedo/micropapillary + ID | High (III) |
| 4681 | Cribriform/papillary + ID | Intermediate (II) |
| 5170 | Comedo + ID | High (III) |
| 6045[b] | Papilloma, comedo + ID | Intermediate (II) |
| 6256 | Cribriform + ID | Low (II) |
| 6384 | Comedo + ID | Intermediate (II) |
| 6457 | Fibroadenosis, comedo + ID | High (III) |

[a] Nuclear grade of DCIS was classified according to van Dongen *et al.* (1992) as low, intermediate or high. For samples in series 2, the grade of the invasive component is given in parentheses when known, and was assessed according to Elston and Ellis (1991). [b] Sample 6045 was classified as comedo DCIS adjacent to invasive, with papilloma present. On the section we obtained there was no DCIS; therefore, only the papilloma and invasive cells were studied (see Table II).

**Table II** Results of A1 studies at chromosome 17q12 – 23; the *BRCA1* gene is known to map between D17S250 and D17S579

| *Case* | *6* | *15* | *34* | *56* | *75* | *106* | *144* | *257* | *1886* | *2281* | *3410* | *3800* | *3805* | *4119* | *4736* | *4753* | | | |
|---|---|---|---|---|---|---|---|---|---|---|---|---|---|---|---|---|---|---|---|
| *Series 1:* | | | | | | | | | | | | | | | | | | | |
| Locus | C12 | MP | C | MP | C12 | Cr12 | C12 | C | Cr12 | C12 | C | CrC | C12 | C | C12 | C12 | | | |
| D17S250 | ●● | – | ○ | – | – | ●○ | ●○ | ● | ○○ | ●● | ● | ●● | ●● | – | ●● | ●● | | | |
| D17S579 | | ○ | ○ | ● | ●○ | | ● | ● | ● | | ○ | | | ○ | | | | | |
| D17S588 | ●○ | ● | ● | ● | ●○ | ●○ | – | ● | ○● | ○○ | ○ | ○● | ●● | – | ●● | – | | | |
| GH | ● | ● | ● | ○ | ●○ | ●○ | ● | ● | | – | ○ | – | | ○ | | – | | | |
| *Series 2:* | | | | | | | | | | | | | | | | | | | |
| Case | 452 | 458 | 593 | 982 | 1141 | 1565 | 2753 | 2822 | 2939 | 2996 | 3041 | 4410 | 4617 | 4681 | 5170 | 6045 | 6256 | 6384 | 6457 |
| Locus | MPI | CrI | Cr I | Cr I | Cr I | B C1 2 I | Cr I | Cr I | Cr I | B C I | Cr I | CI | MPI | P Cr I | C I | B I | Cr I | C I | B C I |
| D17S250 | – | ○● | ●● | ● | ○○ | ○●●†● | ○● | ●● | ○○ | ○●● | ● | ○○ | ●● | ○○● | ●●† | ●○ | – | ○● | ●●● |
| D17S579 | ○○ | | | ●● | ○○ | | | | | | | – | | | ●●† | ●○ | | | – |
| D17S588 | – | ○● | – | – | – | – | ○● | ○○ | ○○ | ○●● | ●●† | – | – | ○● | ●●† | – | ●● | ○● | ●● |
| GH | ○○ | – | ○○ | ●● | | | ○● | – | ○○ | – | – | | ○○ | – | | ●○ | | ○● | |

○, Informative and both alleles retained; ●, Informative and showing allelic imbalance; – , Not informative i.e. homozygous; no symbol, not tested due to lack of further tumour material/sample consistently failed to amplify at that marker; †, different allele retained; C, comedo DCIS; Cr, cribriform DCIS; MP, micropapillary DCIS; P, papillary DCIS; B, benign; I, invasive carcinoma.

**Figure 1** Autoradiographic examples of AI on 17q in cases of DCIS, with or without an associated area of invasive carcinoma. (**a**) AI in the cribriform component of case 106. (**b**) AI with loss of the same alleles in both the comedo and invasive components of case 1565. In contrast, the second area of comedo DCIS from the same tumour shows loss of the other allele. (**c**) AI in the comedo component but not the invasive tumour of case 5170. (**d**) AI in the papilloma but not the invasive component of case 6045. N, normal tissue; C, comedo DCIS; Cr, cribriform DCIS; I, invasive carcinoma; B, papilloma.

breast tumours to date (Futreal *et al*., 1994), although there are recent reports of mutations in sporadic ovarian tumours (Merajver *et al*., 1995). If *BRCA1* is not involved in sporadic breast tumour development this may be because the wild-type product acts at a stage of breast development that precedes sporadic tumour initiation. There are however a number of other genes in the vicinity of *BRCA1* that are possible targets of deletion in sporadic breast tumours, despite the fact that they were eliminated as candidates for *BRCA1*; for example *NM23* and *PHB* (prohibitin) (Leone *et al*., 1991; Sato *et al*., 1992; Royds *et al*., 1993), alterations to both of which have been previously demonstrated in sporadic breast tumours. In addition the c-*erb*B-2 gene maps between D17S250 and D17S579. Overexpression of c-*erb*B-2 has frequently been observed in breast tumours and this is generally thought to be due to gene amplification. A number of the cases of DCIS showing AI at the *BRCA1* region in this study have previously been shown to overexpress c-*erb*B-2 (R Walker, unpublished). However, the patterns of AI observed in these tumours were suggestive of allele loss rather than gain. Therefore, if c-*erb*B-2 is amplified in these cases the amplicon must be on the remaining homologue, indicating the presence of multiple alterations on chromosome 17q in these cases. In addition, two cases were found to show AI at a region independent from *BRCA1*, suggesting a second tumour-suppressor gene on 17q. This has been reported previously by several groups (Cornelis *et al*., 1993; Cropp *et al*., 1993).

The finding that 12 out of 18 cases with both DCIS and invasive tumour show the same pattern of AI in each component suggests that DCIS can progress to invasive carcinoma. That this is not the case for all DCIS is clear from those cases showing distinct alterations in both components. This is complicated further by the heterogeneity present within DCIS. Whatever the target of the AI observed in this study, it is clear from those cases with DCIS and invasive components that its alteration can occur early or late in the development of breast tumours.

Four of the cases reported here contained benign lesions (1565, 2996, 6045 and 6457), and two of these showed AI on 17q. In neither case was Pagetoid spread considered a possibility, and in both cases only epithelial cells were analysed. In case 6457 (fibroadenosis + comedo DCIS + invasive tumour) the same pattern of AI was seen in all three components. However in 6045, AI was only observed in the papilloma and not in the invasive cells (DCIS cells were not present on the sections made available to us and were therefore not studied). The finding of a case that shows AI in a papilloma but not in the associated invasive tumour questions the importance of changes to sequences on 17q for malignant transformation. Alterations to 17q present in the later stages of tumour development might contribute to malignancy by co-operating with other changes such as *TP53* mutations. Their role in benign lesions remains to be elucidated.

**Acknowledgements**
We acknowledge the Cancer Research Campaign for financial support. KEM was in receipt of a Medical Research Council studentship.

### References

BETSILL WL, ROSEN PP, LIEBERMAN PH AND ROBBINS GF. (1978). Intraductal carcinoma: long-term follow-up after treatment by biopsy alone. *JAMA*, **239,** 1863–1867.

BORG A, ZHANG Q-X, JOHANNSSON O AND OLSSON H. (1994). High frequency of allelic imbalance at the BRCA1 region on chromosome 17q in both familial and sporadic ductal breast carcinomas. *J. Natl Cancer Inst.*, **86,** 792–794.

CORNELIS RS, DEVILEE P, VAN VLIET M, KUIPERS-DIJKSHOORN N, KERSENMAEKER A, BARDOEL A, MEERA KHAN P AND CORNELISSE CJ. (1993). Allele loss patterns on chromosome 17q in 109 breast carcinomas indicate at least two distinct target regions. *Oncogene*, **8,** 781–785.

CROPP CS, CHAMPEME M-H, LIDEREAU R AND CALLAHAN R. (1993). Identification of three regions on chromosome 17q in human primary breast carcinomas which are frequently deleted. *Cancer Res.*, **53,** 5617–5619.

DUPONT WD AND PAGE DL. (1985). Risk factors for breast cancer in women with proliferative breast disease. *N. Engl. J. Med.*, **312,** 146–151.

ELSTON CW AND ELLIS IO. (1991). Pathological prognostic factors in breast cancer. I. The value of histological grade in breast cancer: experience from a large study with long-term follow-up. *Histopathology*, **19,** 403–410.

FUTREAL PA, SODERKVIST P, MARKS JR, IGLEHART JD, COCHRAN C, BARRETT JC AND WISEMAN RW. (1992). Detection of frequent allelic loss on proximal chromosome 17q in sporadic breast carcinoma using microsatellite length polymorphisms. *Cancer Res.*, **52,** 2624–2627.

FUTREAL PA, LIU Q, SHATTUCK-EIDENS D, COCHRAN C, HARSHMAN K, TAVTIGIAN S, BENNETT LM, HAUGEN-STRANO A, SWENSEN J, MIKI Y, EDDINGTON K, MCCLURE M, FRYE C, WEAVER-FELDHAUS J, DING W, GHOLAMI Z, SODERKVIST P, TERRY L, JHANWAR S, BERCHUCK A, IGLEHART JD, MARKS J, BALLINGER DG, BARRETT JC, SKOLNICK MH, KAMB A AND WISEMAN R. (1994). BRCA1 mutations in primary breast and ovarian carcinomas. *Science*, **266,** 120–122.

HALL JM, FRIEDMAN L, GUENTHER C, LEE MK, WEBER JL, BLACK DM AND KING M-C. (1992). Closing in on a breast cancer gene on chromosome 17q. *Am. J. Hum. Genet.*, **50,** 1235–1242.

HOGGARD N, BRINTNELL B, HOWELL A, WEISSENBACH J AND VARLEY JM. (1995). Allelic imbalance on chromosome 1 in human breast cancer. II. Microsatellite repeat analysis. *Genes, Chrom. Cancer*, **12,** 24–31.

LEONE A, MCBRIDE OW, WESTIN A, WANG M, ANGLARD P, CROPP CS, LINEHAN MW, REES R, CALLAHAN R, HARRIS C, LIOTTA LA AND STEEG PS. (1991). Somatic allele deletion of *nm23* in human cancer. *Cancer Res.*, **51,** 2490–2493.

LIU E, THOR A, HE M, BARCOS M, LJUNG B-M AND BENZ C. (1992). The HER2(c-*erb*B-2) oncogene is frequently amplified in *in situ* carcinomas of the breast. *Oncogene*, **7,** 1027–1032.

MERAJVER S, PHAM TM, CADUFF RF, CHEN M, POY EL, COONEY KA, WEBER BL, COLLINS FS, JOHNSTON C AND FRANK TS. (1995). Somatic mutations in the *BRCA1* gene in sporadic ovarian tumours. *Nature Genetics*, **9,** 439–443.

MIKI Y, SWENSEN J, SHATTUCK-EIDENS D, FUTREAL PA, HARSHMAN K, TAVTIGIAN S, LIU Q, COCHRAN C, BENNETT LM, DING W, BELL R, ROSENTHAL J, HUSSEY C, TRAN T, MCCLURE M, FRYE C, HATTIER T, PHELPS R, HAUGEN-STRANO A, KATCHER H, YAKUMO K, GHOLAMI Z, SHAFFER D, STONE S, BAYER S, WRAY C, BOGDEN R, DAYANANTH P, WARD J, TONIN P, NAROD S, BRISTOW PK, NORRIS FH, HELVERING L, MORRISON P, ROSTECK P, LAI M, BARRETT JC, LEWIS C, NEUHAUSEN S, CANNON-ALBRIGHT L, GOLDGAR D, WISEMAN R, KAMB A AND SKOLNICK MH. (1994). A strong candidate for the breast, and ovarian cancer susceptibility gene *BCRA1*. *Science*, **266,** 66–71.

MUNN KE, WALKER RA AND VARLEY JM. (1996). Mutation of the *TP53* gene and allelic imbalance at chromosome 17p13 in ductal carcinoma *in situ*, (manuscript in preparation).

MUNN KE, WALKER RA AND VARLEY JM. (1995). Frequent alterations of chromosome 1 in ductal carcinoma *in situ* of the breast. *Oncogene* **10,** 1653–1657.

PAGE DL, DUPONT WD, ROGERS LW AND LANDENBERGER M. (1982). Intraductal carcinoma of the breast: follow-up after biopsy only. *Cancer*, **49,** 751–758.

POLYMEROPOULOS MH, RATH DS, XIAO H AND MERILL CR. (1991). A simple sequence repeat polymorphism at the human growth hormone locus. *Nucleic Acids Res.*, **19,** 689.

PONDER B. (1988). Gene losses in human tumours. *Nature*, **335,** 400–402.

RADFORD DM, FAIR K, THOMPSON AM, RITTER JH, HOLT M, STEINBRUECK T, WALLACE M, WELLS SA AND DONIS-KELLER HR. (1993). Allelic loss on chromosome 17 in ductal carcinoma *in situ* of the breast. *Cancer Res.*, **53,** 2947–2950.

ROSEN PP, BRAUN DW AND KINNE DE. (1980). The clinical significance of pre-invasive breast carcinoma. *Cancer*, **46,** 919–925.

ROYDS JA, STEPHENSON TJ, REES RC, SHORTHOUSE AJ AND SILCOCKS PB. (1993). Nm23 protein expression in ductal carcinoma *in situ* and invasive human breast carcinoma. *J. Natl Cancer Inst.*, **85,** 72–76.

SATO T, SAITO H, SWENSEN J, OLIFANT A, WOOD C, DANNER D, SAKAMOTO T, TAKITA K, KASUMI F, MIKI Y, SKOLNICK M AND NAKAMURA Y. (1992). The human prohibitin gene located on chromosome 17q21 is mutated in sporadic breast cancer. *Cancer Res.*, **52,** 1643–1646.

STRATTON MR, COLLINS N, LAKHANI SR AND SLOANE JP. (1995). Loss of heterozygosity in ductal carcinoma *in situ* of the breast. *J. Pathol.*, **175,** 195–201.

TAVASSOLI FA AND NORRIS HJ. (1990). A comparison of the results of long-term follow-up for atypical intraductal hyperplasia and intraductal hyperplasia of the breast. *Cancer*, **65,** 518–529.

VAN DONGEN JA, HOLLAND R, PETERSE JL, FENTIMAN IS, LAGIOS MD, MILLIS RR AND RECHT A. (1992). Ductal carcinoma *in situ* of the breast; second EORTC consensus meeting. *Eur. J. Cancer*, **28,** 626–629.

WEBER JL, KWITEK AE, MAY PE, WALLACE MR, COLLINS FS AND LEDBETTER DH. (1990). Dinucleotide repeat polymorphisms at the D17S250 and D17S261 loci. *Nucleic Acids Res.*, **18,** 4640.